# Real-World Outcomes of Baricitinib Monotherapy Versus csDMARD Combination Therapy in Rheumatoid Arthritis: A Single-Center Retrospective Analysis of Efficacy, Safety, and Drug Retention

**DOI:** 10.5152/ArchRheumatol.2025.11131

**Published:** 2025-09-01

**Authors:** Mete Kara, Gülay Alp, Haluk Cinakli

**Affiliations:** 1 Department of Rheumatology, University of Health Sciences, İzmir Bozyaka Education and Research Hospital, İzmir, Türkiye; 2 Division of Rheumatology, Department of Internal Medicine, Uşak University School of Medicine, Uşak, Türkiye; 3 Department of Rheumatology, Kırklareli Educational and Research Hospital, Kırklareli, Türkiye

**Keywords:** Antirheumatic-agents, baricitinib, combination, monotherapy, rheumatoid arthritis

## Abstract

**Background/Aims:**

This study compared the effectiveness, adverse effects (AEs), and drug retention rates of baricitinib (BARI) monotherapy versus combination therapy in rheumatoid arthritis (RA) patients.

**Materials and Methods:**

In this single-center retrospective observational study, 140 RA patients were analyzed, with 50 receiving monotherapy and 90 receiving BARI combination therapy. Demographics, disease characteristics, treatment details, and AEs were recorded. Clinical outcomes were compared between the groups, including disease activity, assessed by the Disease Activity Score in 28 Joints with C-reactive Protein (DAS28-CRP), Simplified Disease Activity Index (SDAI), and Clinical Disease Activity Index (CDAI), as well as functional status and drug survival.

**Results:**

Baricitinib monotherapy and BARI combination groups had similar baseline characteristics. Both groups showed significant improvements in disease activity, with no difference in final DAS28-CRP, SDAI, or CDAI scores. A higher proportion of BARI monotherapy patients achieved low disease activity on SDAI and CDAI. Adverse effects rates were similar between groups, though serious AEs were slightly more common in combination therapy (*P* = .044). This study found no significant difference in drug survival between monotherapy and combination therapy. In multivariate analysis, higher initial steroid dosage (hazard ratio (HR) = 1.149, *P* = .030), prior use of 2 or more biologic disease-modifying antirheumatic drugs (HR = 2.825, *P* = .002), and younger age (HR = 0.957, *P* = .001) were significant predictors of BARI treatment discontinuation.

**Conclusion:**

This study suggests that BARI monotherapy offers comparable efficacy, safety, and retention to the BARI combination in RA treatment. It provides an effective alternative for patients who find it inconvenient to use conventional synthetic disease-modifying antirheumatic drugs.

**Main Points**

- Baricitinib monotherapy was as effective and safe as csDMARD combination therapy in RA.
- Higher steroid dose and multiple prior bDMARDs increased the risk of discontinuation.
- Older age reduced discontinuation risk; BARI plus leflunomide was well tolerated.

## Introduction

Rheumatoid arthritis (RA) is a common systemic inflammatory disease affecting joints and extra-articular structures. The specific pathogenesis of RA is still unclear, and curative treatment does not currently appear possible. The main goal of RA treatment is currently to control and alleviate the disease.^1^ Appropriate use of disease-modifying antirheumatic drugs (DMARDs) allows RA to be controlled. Significant progress has been made in treating RA with biologic DMARDs (bDMARDs), which have been available for the past few decades, and targeted synthetic DMARDs (tsDMARDs), which have become increasingly popular recently. Despite newer treatment options, methotrexate (MTX) remains the first-line therapy, while leflunomide (LEF) has shown comparable efficacy and can be used as monotherapy or in combination.^2-4^

Janus kinase inhibitors (JAKi) are in the group of tsDMARDs that can be as effective as bDMARDs and have specific AEs. Baricitinib (BARI) is a JAK1 and JAK2 inhibitor used to treat RA. Baricitinib positively affects disease activity, function, and structural damage in RA patients^5^ and is an approved treatment for RA when conventional synthetic DMARD (csDMARD) and bDMARD drugs have failed.^6,2^ It may be used with MTX, LEF, or other DMARD therapies or as monotherapy. Real-life data have been reported showing that BARI is used effectively as monotherapy in cases of MTX intolerance with generally high drug persistence rates, without showing any new safety signals.^7^ The efficacy of BARI has been studied in randomized controlled trials^8^ and several real-life studies in patients with active RA as monotherapy and in combination with MTX.^9,10^ Baricitinib monotherapy is at least as effective as combination therapy with MTX in randomized controlled trials.^8^ Multicenter observational cohort confirmed BARI’s efficacy and safety profile in bDMARD-naive RA patients and demonstrated improved drug survival in patients with bDMARD-naive and seropositive.^9^

There are limited studies on using BARI on the efficacy and drug retention of BARI as monotherapy versus in combination with a csDMARD in real life. These studies have shown the effect of BARI monotherapy on disease activity and functionality, and BARI has generally been used with MTX in these studies.^11,12^

However, to the authors’ knowledge, there is no data in the literature regarding its combination with other csDMARDs, except for a case series published for LEF in 5 patients.^13^ Although the efficacy of BARI is well established, further real-world studies are needed to clarify its safety profile, drug retention, and patterns of use as monotherapy or in combination with csDMARDs. This is further complicated by the different approaches of clinicians in different societies for a disease without a standardized treatment.

The primary objective of this study was to evaluate the drug survival time (i.e., drug retention rate) of BARI monotherapy compared to combination therapy with csDMARDs in patients with RA. Secondary objectives included the assessment of treatment efficacy using clinical composite scores (DAS28-CRP, SDAI, and Clinical Disease Activity Index [CDAI], the achievement of low disease activity (LDA), and the incidence of AEs).

## Methods

This retrospective observational study was conducted between May 2021 and January 2023 at the Department of Rheumatology at the University of Health Sciences İzmir Bozyaka Education and Research Hospital. It included patients with more than 6 months of follow-up diagnosed with RA according to the 2010 Rheumatoid Arthritis Classification Criteria by the American College of Rheumatology/European Alliance of Associations for Rheumatology (EULAR).^14^

The demographic characteristics of the patients, their usage of medications, previous medications, smoking status, and comorbidity data were noted from the electronic patient registration system by the study physician (M.K.), who was responsible for data entry and verification. All comorbidity data have been processed by the classification of diseases described by Charlson.^15^ Patients were routinely monitored every 3 months, and data were collected at treatment initiation and final follow-up visits. The Disease Activity Score C-Reactive Protein (DAS28-CRP) was used to evaluate disease activity for RA.^16^ In addition to DAS-28 CRP, Simplified Disease Activity Index (SDAI), CDAI, and Health Assessment Questionnaire disability index (HAQ DI) scores were also assessed at the beginning and last visit of the treatment. Low disease activity was defined as DAS28-CRP ≤ 3.2, SDAI ≤ 11, or CDAI ≤ 10.

The treatment of the patients was decided according to the evaluation of the monitoring physician, considering age, comorbidity, renal failure, drug compliance, initial disease activity and risk factors, and current guidelines. In cases of insufficient response or intolerance after csDMARD, patients who were started on BARI treatment were treated with BARI monotherapy or in combination with csDMARD according to the physician’s evaluation. The patients’ glucocorticoid (GC) doses during this period were also recorded. Patients who used these treatments regularly for at least 6 months were included in the evaluation of efficacy and AEs. Adverse effects were registered by the physician at each visit and coded according to the Medical Dictionary for Regulatory Activities (MedDRA) coding system.^17^ The efficacy of BARI monotherapy and BARI csDMARD combination therapy was compared in terms of disease activity, AEs, and drug retention.

### Statistical Analysis

Both visual (histogram and probability plots) and analytical methods (Kolmogorov–Smirnov test) were used to check whether the variables had a normal distribution. The categorical variables were presented as percentages and frequencies, and the continuous variables were expressed as mean ± SD for normally distributed data, and as median with interquartile range (IQR) for non-normally distributed data. Categorical variables were analyzed using the chi-square test when the expected frequency in each cell was ≥5, and Fisher’s exact test was used otherwise. An independent samples *t*-test was used to compare 2 independent groups with normal distribution, and the Mann–Whitney *U* test was used to compare 2 groups of non-normality distributed data. Drug persistence was evaluated using Kaplan–Meier analysis, and drug survival rates between the 2 groups were compared using the log-rank test. Then, Cox analysis was performed to evaluate the predictive factors of drug discontinuation rate and to determine which risk factors affected treatment continuity. All covariates that were statistically significant (*P* < .05) or borderline significant (*P* < .1 and > .05) in the bivariate analysis and those considered to be clinically significant were included in the multivariate Cox regression analysis by backward elimination of the variables. The level of statistical significance level was accepted as *P* < .05. The Statistical Package for the Social Sciences (IBM SPSS) 25.0 (IBM SPSS Corp.; Armonk, NY, USA) was used for the analysis of the collected data.

As this was a retrospective study including all eligible patients during the study period, no a priori sample size calculation was performed. However, a post hoc power analysis based on 43 treatment discontinuation events among 140 patients, assuming a hazard ratio of 2.825 and a significance level of α = 0.05, showed an estimated power of approximately 85%, indicating sufficient statistical power to detect clinically meaningful effects.

### Ethics Approval

Ethics committee approval for this study was received locally from the University of Health Sciences İzmir Bozyaka Education and Research Hospital (decision dated January 25, 2023, numbered 09). The study was conducted following the principles of the Declaration of Helsinki. Since this study was planned retrospectively, approval was obtained from the ethics committee without obtaining informed consent from the patients.

## Results

The total number of included RA patients was 140, out of which 50 received BARI monotherapy and 90 received BARI combination therapy. The demographic and clinical characteristics of the 2 groups are presented in Table 1.

In this study, 50 patients received BARI monotherapy, and 90 received BARI combination therapy. The groups were comparable in age, age at diagnosis, and female proportion. Disease duration, follow-up period, smoking status, and seropositivity (Rheumatoid factor (RF) and/or anti-citrullinated protein antibodies (ACPA) positivity) rates showed no significant differences between groups. Initial prednisolone dose was slightly higher in the combination group (6.4 ± 2.2 mg vs. 5.8 ± 1.7 mg, *P* = .094), with a significant difference in final prednisolone dose (2.7 ± 2.4 mg vs. 1.5 ± 2.1 mg, *P* = .003).

In terms of treatment regimens, 61 patients (67.7%) were receiving combination therapy with MTX, 26 (28.9%) with LEF, while 2 were treated with sulfasalazine, and 1 hydroxychloroquine. A history of previous bDMARD use was reported in 26% of the monotherapy group and 40% of the combination therapy group (*P* = .061). The mean BARI dosage was similar across groups (3.9 ± 0.2 vs. 3.8 ± 0.5, *P* = .226) (Table 1).

In this study, initial DAS28-CRP scores were significantly lower for the BARI monotherapy group compared to the combined therapy group, with an average initial DAS28-CRP of 5.44 versus 5.59 (*P* = .003). However, the final DAS28-CRP scores showed no significant difference between groups, indicating similar efficacy at the study endpoint. Additionally, no significant differences were found in the final SDAI and CDAI scores. The percentage of patients achieving LDA was higher in the BARI monotherapy group across SDAI (88% vs. 70%, *P* = .016) and CDAI (90% vs. 70%, *P* = .007), although no significant difference was observed in DAS28-CRP LDA rates. Mean changes in DAS28-CRP, SDAI, and CDAI scores from baseline were similar between the groups. Health Assessment Questionnaire scores also showed no significant differences (Table 2).

**Figure 1.**
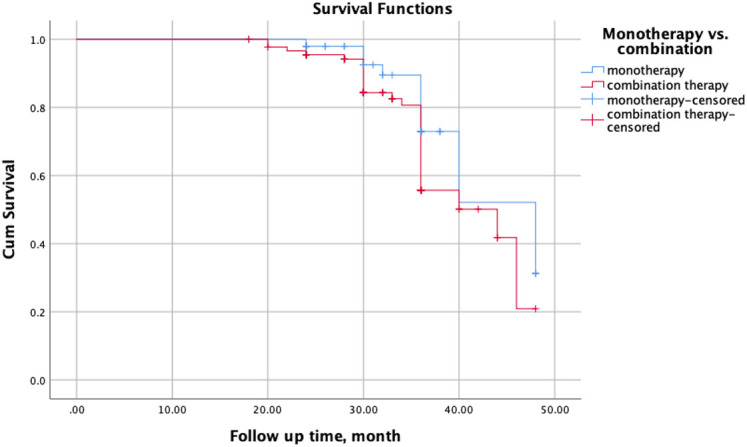
Kaplan–Meier curves showing drug survival of baricitinib in patients receiving monotherapy versus combination therapy with csDMARDs.

Adverse effects were overall comparable between the BARI monotherapy and combination therapy groups (18% vs. 18.9%, *P* = .897). There were no statistically significant differences between groups in terms of hematologic, renal, infectious, or metabolic events (Table 3). However, the cumulative incidence of serious AEs was significantly higher in the combination therapy group (5.6% vs. 2%, pp = 0.044). Although discontinuation due to AEs and overall treatment discontinuation were more frequent in the combination group, these differences did not reach statistical significance. This study found no significant difference in drug survival between BARI monotherapy and combination therapy groups (long rank test = 0.116) (Figure 1). The median survival time was 48 months (95% CI: 40.07-55.92) for monotherapy and 44 months (95% CI: 32.61-55.38) for combination therapy. Although the monotherapy group had a slightly longer median survival, the overlapping confidence intervals suggest no statistically significant difference between the 2 groups.

In analyzing factors associated with BARI treatment discontinuation due to lack of effectiveness or AEs, bivariate Cox regression identified several significant predictors. A higher initial steroid dosage increased the risk of discontinuation, as did prior use of bDMARDs, particularly when patients had received 2 or more. Additionally, higher DAS28-CRP scores at baseline were linked to an increased risk. Age was protective, with older patients exhibiting a lower risk of discontinuation (*P* = .002). In the multivariate analysis, initial steroid dosage remained a significant risk factor (HR = 1.149, 95% CI: 1.013-1.303, *P* = .030), as did having a history of at least 2 prior bDMARDs (HR = 2.825, 95% CI: 1.462-5.458, *P* = .002). Age continued to show a protective effect (HR = 0.957, 95% CI: 0.932-0.958, *P* = .001), indicating a reduced likelihood of discontinuation with increasing age (Table 4).

For patients receiving BARI monotherapy, initial steroid dosage (*P* = .012) and prior use of 2 or more bDMARDs (*P* = .009) were significant predictors of treatment discontinuation in the bivariate analysis, while older age was found to be protective (*P* = .008). In the multivariate model, these associations persisted, with initial steroid dosage (HR = 1.149, 95% CI: 1.013-1.303, *P* = .030) and prior 2 or more bDMARD use (HR = 5.517, 95% CI: 1.326-22.958, *P* = .019) remaining significant, and age continuing to demonstrate a protective effect (HR = 0.933, 95% CI: 0.884-0.984, *P* = .011) (Table 5).

In the multivariate Cox regression analysis, 3 variables were independently associated with baricitinib treatment discontinuation. Increasing age was associated with a reduced risk of treatment discontinuation (HR = 0.969, 95% CI: 0.940-1.000, *P* = .049). In contrast, higher initial steroid dosage (HR = 1.191, 95% CI: 1.032-1.374, *P* = .017) and prior failure of ≥2 bDMARDs (HR = 2.431, 95% CI: 1.126-5.250, *P* = .024) were associated with a significantly increased risk of treatment discontinuation (Table 6).

## Discussion

In this study, it was demonstrated that BARI monotherapy was as effective as combination therapy with csDMARDs in terms of efficacy, safety, and drug retention, with no significant difference in drug survival between the 2 approaches. Factors such as higher initial steroid dosage and prior use of 2 or more bDMARDs emerged as significant predictors of treatment discontinuation, while age shows a protective effect on treatment discontinuation.

In the RA-BEGIN study, BARI was evaluated in terms of disease activity, patient functional responses, and slowing of radiographic progression in comparison to MTX, both as monotherapy and in combination. Baricitinib monotherapy showed that BARI plus MTX was similar in terms of clinical and functional responses and that both were significantly better than MTX alone, although radiographic slowing was only significant in combination therapy versus MTX monotherapy. Similarly, in this study, the reduction in disease activity and improvement in functional responses were similar in both groups in BARI monotherapy and combination therapy. Considering that patients receiving BARI in real life had previously been unresponsive or intolerant to MTX and/or other csDMARDs, these results will gain more significance for daily practice. This study did not perform radiographic evaluation as in the RA-BEGIN study.^8^

In the RA-BEYOND study, as a continuation of the RA-BEGIN study, all patients were switched to BARI monotherapy and followed up. According to the physician’s decision, MTX was added to the vast majority, and other csDMARDs were added to a small number during their follow-up. In this study, the disease activities of patients to whom MTX was added were higher than those to whom MTX was not added, and a greater decrease in disease activity was observed in patients to whom MTX was added.^18^ Although the disease activity of patients receiving BARI combination therapy was higher than monotherapy in this study, Δ DAS28, which shows the decrease in disease activity at the end of treatment, was similar in both groups. In this study, in combination use, there was also use of other csDMARDs, especially LEF, in addition to MTX, and in real-life data where these drugs were used in combination, both groups were found to be similar in terms of disease activity, functional change, and AEs.

Real-life data from different European countries have confirmed the efficacy and safety of BARI.^10,12^ In a prospective study comparing BARI monotherapy with MTX, BARI monotherapy yielded similar results to MTX in terms of efficacy and drug retention.^7^ In this study, not all those in the combined group received MTX, and similar results in BARI monotherapy and combined use can be stated for other csDMARD uses.

Janus kinase inhibitors use has been associated with an increased risk of herpes zoster and cardiovascular events, venous thromboembolic events, and malignancies.^19^ Baricitinib has shown AEs consistent with other JAKi and bDMARDs, but long-term data up to 9.3 years identified no new safety signals.^20^ In this study, AEs were similar in both groups, and serious AEs were more common in the combined group than in the monotherapy group (*P* = .044). This may be due to the higher initial disease activity of patients receiving combined therapy, concomitant medications, or possible polypharmacy. Although polypharmacy was not evaluated in this study, the effects of polypharmacy on efficacy and safety in RA patients may have negative effects.^21^ Since all DMARDs are used chronically in patients with RA, the long-term safety profile requires continuous monitoring and evaluation. In this study, the average patient follow-up period was 32.7 months, and longer follow-up may be needed in terms of the development of AEs such as major cardiovascular events and malignancy.

A previously published case series explored the potential drug interactions between BARI and LEF, reporting thrombocytosis in all 5 patients studied. Although no thrombotic events occurred, an increase in infections was observed, which resolved within 3-6 months after discontinuing both medications. This interaction was hypothesized to be mediated by the renal organic anion transporter-3 inhibitory effect of LEF, potentially altering BARI plasma levels.^13^ However, in the current study, a subgroup analysis of 26 patients treated with the LEF combination revealed no instances of thrombocytosis or thrombotic complications. These findings suggest that the safety profile of this combination may be favorable under real-world conditions, although close monitoring remains essential.

The impact of seropositivity on treatment discontinuation remains inconclusive. In this study, there was no association between seropositivity and drug discontinuation. Similarly, a previous study reported that seropositivity did not influence JAKi treatment retention.^22^ However, in contrast, a multicenter real-world dataset demonstrated that both RF and ACPA positivity reduced the risk of BARI discontinuation due to ineffectiveness. Additionally, the same study found that BARI treatment improved adherence in bDMARD-naive seropositive RA patients.^9^ These discrepancies across studies highlight the need for further research to clarify the role of seropositivity in treatment outcomes. Differences in patient populations, treatment settings, and study designs likely contribute to these variations, underscoring the importance of larger, multicenter studies.

In this study, BARI drug survival and remission rates of the monotherapy and combination groups were not different. Published studies have consistently reported that RA disease duration does not significantly influence treatment retention, likely due to effective disease management protocols established in clinical practice.^23^ In alignment with these findings, this study also did not observe a significant association between RA disease duration and treatment discontinuation due to lack of effectiveness and AEs. This may reflect advances in RA management, particularly the availability of tsDMARDs like BARI for patients with long-standing diseases.

In this study, Cox regression analysis showed that when all drug discontinuation events were evaluated together due to the small number of events, a history of ≥2 prior bDMARD failures was significantly associated with drug discontinuation (HR: 2.82; *P* = .002). This finding aligns with evidence suggesting that treatment outcomes tend to worsen with increasing lines of therapy. Previous studies have demonstrated that patients who fail multiple bDMARDs exhibit lower drug retention rates and reduced efficacy, likely due to drug resistance or cumulative disease burden.^23^ These observations underscore the importance of prioritizing effective therapies, such as JAKi, early in the treatment algorithm for these patients.

Studies examining JAKi discontinuation due to AEs, older age (>65 years), and female gender have been identified as potential patient-related factors associated with an increased risk of treatment discontinuation.^24^ Interestingly, in contrast to existing literature, this study found that older age was associated with a protective effect against BARI discontinuation. Several factors may explain this discrepancy. Elderly patients often present with less aggressive disease due to immunosenescence, tend to prioritize treatment stability and generally exhibit better adherence. Additionally, physicians may manage older patients more cautiously, optimizing doses and closely monitoring AEs to minimize risks. Furthermore, mild AEs may be attributed to aging rather than the medication itself, potentially reducing the likelihood of treatment discontinuation. These factors, combined with BARI’s favorable pharmacodynamic profile, may contribute to improved drug retention in this population. These findings, derived from patients receiving baricitinib in combination with csDMARDs, suggest that younger patients with a history of multiple prior bDMARD failures and higher baseline steroid needs may be more prone to treatment discontinuation.

The findings provide indirect evidence that LEF in combination with BARI may be a safe alternative for patients unable to use MTX, with no observed increase in AEs. However, several limitations should be acknowledged. First, the findings may not be generalizable to broader patient populations, such as biologic-naïve or treatment-resistant individuals, due to potential selection bias inherent in real-world data. Although a post hoc power analysis based on all treatment discontinuation events indicated sufficient power for overall analyses, the number of AE-related discontinuations alone was too small to support reliable subgroup analyses. The limited number of patients receiving LEF in combination with BARI (n = 26) restricts definitive conclusions about its safety or efficacy, supporting only indirect evidence for its potential as an alternative to methotrexate. The retrospective design may have led to incomplete or biased documentation of AEs. While the mean follow-up was 32.7 months, it is acknowledged that this duration may still be insufficient to detect some long-term AEs, such as cardiovascular complications or malignancies, which typically emerge after prolonged exposure. Finally, radiological outcomes were not evaluated, preventing an assessment of whether the combination therapy provides superior protection against disease progression, as suggested by previous research.

In conclusion, this study demonstrated that BARI monotherapy is as effective and safe as combination therapy with csDMARDs in RA patients. Notably, the absence of increased AEs in those receiving combination therapy with LEF supports its use as an alternative for patients who cannot tolerate MTX. Drug survival was influenced by prior bDMARD use and initial steroid dosage, whereas seropositivity and other baseline characteristics showed no significant association. These findings highlight the clinical utility of BARI monotherapy, particularly for patients with contraindications to csDMARDs, reinforcing its role as a viable treatment option in routine practice.

## Figures and Tables

**Table 1.** Comparison of Demographic Disease Characteristics and Treatments of Patients Receiving Baricitinib Monotherapy and Combination Therapy

Variables	BARI Monotherapy (n = 50)	BARI Combine Therapy (n = 90)	*P*
Age, years, mean (SD)	49.1 (11.5)	49.3 (12.3)	.921
Age at diagnosis, years, mean (SD)	44.2 (11.1)	44.8 (12)	.776
Sex, female, n (%)	30 (60)	51 (56.7)	.702
Disease duration, years, median (IQR)	3 (4)	3 (3)	.736
Follow-up time, months, mean (SD)	33.3 (7.2)	32.3 (6.2)	.427
Smoking status, current, n (%)	16 (32)	23 (25.6)	.415
Seropositivity, n (%)	38 (76)	60 (66.7)	.248
RF positivity, n (%)	32 (64)	57 (63.3)	.937
ACPA positivity, n (%)	30 (60)	45 (50)	.256
At least 1 comorbidity, n (%)	20 (40)	38 (42.2)	.798
Charlson comorbidity index, median (IQR)	0 (1)	0 (1)	.525
Initial prednisolone dosage, mg, mean (SD)	5.8 (1.7)	6.4 (2.2)	.094
Final prednisolone dosage, mg, mean (SD)	1.5 ( 2.1)	2.7 (2.4)	**.003**
Δ prednisolone dosage, mean (SD)	4.28 (1.99)	3.64 (2.65)	**.111**
Barisitinib dosage, mean (SD)	3.9 (0.2)	3.8 (0.5)	.226
bDMARDs experienced (%)	13 (26)	36 (40)	.061
One previous bDMARD failed (%)	12 (24)	35 (38.9)	.074
Two previous bDMARDs failed (%)	3 (6)	14 (15.6)	.097
≥ 3 previous bDMARDs failed (%)	1 (2)	4 (4.4)	.655

IQR is calculated as the difference between Q3 and Q1.

ACPA, anti-cyclic citrullinated peptide antibody; bDMARDs, biological disease-modifying antirheumatic drugs; DAS28-CRP, Disease Activity Score in 28 joints based on the C-reactive protein; IQR, Interquartile range; RF, Rheumatoid factor; Δ, Delta.

Bold values indicate statistically significant results (*P* < 0.05).

**Table 2.** Comparison of Baricitinib Monotherapy and Combination Therapy in Terms of Efficacy and Functionality

Variables	BARI Monotherapy (n = 50)	BARI Combine therapy (n = 90)	*P*
Initial DAS28-CRP, mean (SD)	5.44 (0.27)	5.59 (0.34)	**.003**
Initial SDAI, mean (SD)	28.3 (7.7)	28.4 (1.7)	.743
Initial CDAI, mean (SD)	26.6 (5.7)	26.8 (3)	.716
Initial HAQ, mean (SD)	1.6 (0.13)	1.6 (0.17)	.214
Final DAS28-CRP, mean (SD)	2.59 (0.83)	2.73 (0.7)	.228
DAS28-CRP, LDA, n (%)	39 (78)	62 (68.9)	.249
Δ DAS28-CRP, mean (SD)	2.92 (0.85)	2.85 (0.65)	.592
Final SDAI, mean (SD)	6.07 (4.88)	6.96 (5.07)	.313
SDAI LDA, n (%)	44 (88)	63 (70)	**.016**
Δ SDAI, mean (SD)	22.2 (5.3)	21.5 (5.12)	.409
Final CDAI, mean (SD)	5.3 (4)	6.61 (5.05)	.110
CDAI LDA, n (%)	45 (90)	63 (70)	**.007**
Δ CDAI, mean (SD)	21.8 (6.01)	20.2 (4.97)	.107
Final HAQ, mean (SD)	1.18 (0.17)	1.21 ( 0.2)	.472
Δ HAQ, mean (SD)	0.45 (0.11)	0.46( 0.11)	.596

CDAI, Clinical Disease Activity Index score; DAS28-CRP, Disease Activity Score in 28 joints based on the C-reactive protein; HAQ, Health Assessment Questionnaire disability index; LDA, low disease activity; SDAI, Simplified Disease Activity Index score; Δ, Delta.

Bold values indicate statistically significant results (*P* < 0.05).

**Table 3.** Comparison of Baricitinib Monotherapy and Combination Therapy in Terms of Adverse Effects and Drug Discontinuation

Variables, n (%)	BARI Monotherapy (n = 50)	BARI Combine Therapy (n = 90)	*P*
Adverse effect	9 (18)	17 (18.9)	.897
Anemia, present	1 (2)	1 (1.1)	1
Leukopenia , present	4 (8)	3 (3.3)	.248
Renal dysfunction, present	1 (2)	1 (1.1)	1
Hyperlipidemia, present	2 (4)	2 (2.2)	.617
Creatine kinase elevation	2 (4)	1 (1.1)	.290
Thrombosis is present	0	1 (1.1)	1
Infection	0	5 (5.6)	.160
Malignancy	0	1 (1.1)	1
Herpes zoster	1 (2)	1 (1.1)	1
Serious adverse effects	1 (2)	5 (5.6)	**.044**
Discontinuation related to adverse effect	2 (4)	5 (5.6)	.421
Discontinuation related to ineffective	11 (22)	24 (26.7)	.541
Discontinuation related to all reason	13 (26)	30 (33.3)	.367

BARI, baricitinib.

Bold values indicate statistically significant results (*P* < 0.05).

**Table 4.** Cox Proportional Hazard Analysis for Baricitinib Treatment Discontinuation Risk Factors Due to Lack of Effectiveness and Adverse Events

Covariate	Bivariate Analysis		Multivariate Analysis	
	*P*	HR (95% CI)	*P*	HR (95% CI)
Age, years	.002	0.960 (0.936-0.985)	**.001**	**0.957 (0.932-0.958)**
Sex, female	.420	0.781 (0.428-1.425)		
Smoking status, current	.782	0.904 (0.443.-1.845)		
Initial steroid dosage	.012	1.175 (1.036-1.332)	**.030**	**1.149 (1.013-1.303)**
Initial DAS28 CRP	.045	2.260 (1.017-5.023)		
Previous bDMARD (≥1)	.025	2.042 (1.094-3.812)		
Previous bDMARD(≥2)	.001	3.142 (1.592-6.202)	**.002**	**2.825 (1.462-5.458)**
Combination vs. monotherapy	.185	1.579 (0.804-3.102)		
Methotrexate usage	.204	1.485 (0.806-2.734)		
Leflunomide usage	.752	1.134 (0.512-2.469)		
Disease duration (years)	.306	0.953 (0.870-1.045)		
Seropositivity (RF or/and ACPA), present	.860	1.063 (0.540-2.090)		
At least 1 comorbidity present	.337	0.744 (0.406-1.362)		
Health Assessment Questionnaire	.118	4.255 (0.693-26.135)		

ACPA, anti-cyclic citrullinated peptide antibody; bDMARDs, biological disease-modifying antirheumatic drugs; DAS28-CRP, Disease Activity Score in 28 joints based on the C-reactive protein; RF, rheumatoid factor.

Bold values indicate statistically significant results (*P* < 0.05).

**Table 5.** Cox Proportional Hazard Analysis for Barisitinib Monotherapy Treatment Discontinuation Risk Factors Due to lack of Effectiveness and Adverse Events

Covariate	Bivariate Analysis		Multivariate Analysis	
	*P*	HR (95% CI)	*P*	HR (95% CI)
Age, years	.008	0.929 (0.880-0.981)	**.011**	**0.933 (0.884-0.984)**
Sex, female	.764	0.846 (0.284-2.521)		
Smoking status, current	.665	1.290 (0.408-4.074)		
Initial steroid dosage, mg	.012	1.175 (1.036-1.332)	**.030**	**1.149 (1.013-1.303)**
Initial DAS28 CRP	.129	2.870 (0.638-12.905)		
Previous bDMARD (≥1)	.146	2.253 (0.753-6.736)		
Previous bDMARD (≥2)	.009	6.306 (1.575-25.276)	**.019**	**5.517 (1.326-22.958)**
Disease duration (years)	.864	0.989 (0.867-1.128)		
Seropositivity (RF or/and ACPA), present	.866	0.903 (0.276-2.594)		
Comorbidity, present	.337	0.744 (0.406-1.362)		
Health Assessment Questionnaire	.761	2.056 (0.693-212)		

ACPA, anti-cyclic citrullinated peptide antibody; bDMARDs, biological disease-modifying antirheumatic drugs; DAS28-CRP, Disease Activity Score in 28 joints based on the C-reactive protein; RF, rheumatoid factor.

Bold values indicate statistically significant results (*P* < 0.05).

**Table 6.** Cox Proportional Hazard Analysis for Risk Factors of Barisitinib Combination Treatment Discontinuation Due to Lack of Effectiveness and Adverse Events

Covariate	Bivariate Analysis		Multivariate Analysis	
	*P*	HR (95% CI)	*P*	HR (95% CI)
Age, years	.068	0.972 (0.943-1.001)	**.049**	0.969 (0.940-1000)
Sex, female	.442	0.754 (0.367-1.549)		
Smoking status, current	.553	0.747 (0.284-1.963)		
Inıtal steroid dosage, mg	.012	1.175 (1.036-1.332)	**.017**	1.191 (1.032-1.374)
Initial DAS28 CRP	.131	2.065 (0.806-5.290)		
Previous bDMARD (≥1)	.083	1.949 (0.917-4.141)		
Previous bDMARD (≥2)	.012	2.634 (1.239-5.599)	**.024**	2.431 (1.126-5.250)
Methotrexate usage	.821	1.095 (0.498-2.409)		
Leflunomide usage	.750	0.876 (0.388-1.979)		
Disease duration (years)	.259	0.953 (0.817-1.056)		
Seropositivity (RF or/and ACPA), present	.746	1.146 (0.503-2.609)		
At least 1 comorbidity present	.546	0.800 (0.388-1.649)		
Health Assessment Questionnaire	.118	4.255 (0.693-26.135)		

ACPA, anti-cyclic citrullinated peptide antibody; bDMARDs, biological disease-modifying antirheumatic drugs; DAS28-CRP, Disease Activity Score in 28 joints based on the C-reactive protein; RF, rheumatoid factor.

Bold values indicate statistically significant results (*P* < 0.05).

## Data Availability

The data that support the findings of this study are available on request from the corresponding author.
